# Comment on: Targeting the HGF/c-MET pathway in advanced pancreatic cancer: a key element of treatment that limits primary tumour growth and eliminates metastasis

**DOI:** 10.1038/s41416-020-1003-7

**Published:** 2020-07-28

**Authors:** Pei Pei Che, Alessandro Gregori, Godefridus J. Peters, Max Dahele, Peter Sminia, Elisa Giovannetti

**Affiliations:** 1 Department of Radiation Oncology, Amsterdam University Medical Centers, VU University, Cancer Center Amsterdam, Amsterdam, The Netherlands; 2 Department of Medical Oncology, Amsterdam University Medical Centers, VU University, Cancer Center Amsterdam, Amsterdam, The Netherlands; 3 Department of Biochemistry, Medical University of Gdansk, Gdańsk, Poland; 4 Cancer Pharmacology Lab, AIRC Start-Up Unit, Fondazione Pisana per la Scienza, Pisa, Italy

**Keywords:** Drug discovery, Cancer

With great interest we have read the article from Xu et al.^1^ about targeting of the HGF/c-MET pathway using an advanced pancreatic cancer experimental model. In particular, we were intrigued by the triple combination of gemcitabine with HGF and c-MET inhibitors, which was found to be the most effective in both reducing the size of the primary tumour and eliminating metastases. The mechanistic deepening of this study demonstrates the combinatorial therapeutic action. While gemcitabine is cytotoxic both on the primary tumour, i.e. the decrease in the number of potentially metastasising cancer cells, and on a, however non-significant, reduction of metastasis, both inhibitors act mainly on EMT cells to further diminish dissemination of cancer cells and by decreasing cancer stemness. The data in this model yield great clinical perspective, and some key points should be further discussed.

We agree with the authors that the AsPC-1 cell line resembles a model for advanced pancreatic cancer, and that the use of other commercially available cell lines would give similar results in terms of pancreatic stellate cell-induced tumour progression. However, as suggested also by Xu and collaborators, tumour sensitivity to gemcitabine could significantly affect the efficacy of the triple treatment. Indeed, the efficacy of the combination over time should preferably be assessed in experimental models with engrafted intrinsically gemcitabine-resistant cell lines and on cell lines showing acquired resistance to gemcitabine due to prolonged exposure. Notably, these models might also acquire a differential sensitivity to other drugs, and we recently showed that xenografts from a gemcitabine-resistant clone of Panc-1 cells were highly sensitive to nab-paclitaxel.^2^

The expression level of c-MET in tumour specimens from patients is a clinically relevant issue. We observed both relatively high and low expression levels of c-MET in patient-derived cancer cells.^3^ Furthermore, high c-MET expression has been correlated with a low overall survival rate.^4^ While the triple combination could have clinical potential for a subset of patients with high c-MET expression, it is highly interesting to investigate if this triple combination would be evenly effective in pancreatic cancer cells with a relatively low c-MET expression. Also, it would be useful to discuss the interaction of this novel treatment combination with irradiation, as suggested by previous data.^4^

Our last concern regards the interesting results on collagen deposition after HGF/c-Met inhibition. Although pancreatic stellate cells are mostly responsible for collagen deposition, there is evidence that also PDAC cells participate in this process by means of contractile force generation which in turn promote remodelling and secretion of extracellular matrix proteins.^5^ To our opinion, the authors’ finding that HGF plus c-MET inhibition is the only effective treatment indicates that targeting both tumour and stromal cells is required to block collagen deposition. Nevertheless, to date, no studies reported the ability of gemcitabine to increase tumour collagen content. In that respect, it is remarkable that the triple therapy had no effect on collagen deposition inhibition. Given the importance of extracellular matrix proteins in pancreatic cancer, we would encourage further investigation of this phenomenon.

## Data Availability

All data generated or analysed during this study are included in this published article.
